# SCO-spondin oligopeptide inhibits angiogenesis in glioblastoma

**DOI:** 10.18632/oncotarget.20837

**Published:** 2017-09-12

**Authors:** Romain Bibes, Stéphane Gobron, François Vincent, Carole Mélin, Nicolas Vedrenne, Aurélie Perraud, Francois Labrousse, Marie-Odile Jauberteau, Fabrice Lalloué

**Affiliations:** ^1^ EA3842 Cellular Homeostasis and Diseases, University of Limoges, Faculty of Medicine, 87025 Limoges Cedex, France; ^2^ Neuronax, Biopôle Clermont-Limagne, 63360 Saint-Beauzire, France; ^3^ Limoges University Hospital, Department of Physiological Functional Investigation, 87042 Limoges Cedex, France; ^4^ Limoges University Hospital, Department of Digestive Surgery, 87042 Limoges Cedex, France; ^5^ Limoges University Hospital, Department of Pathology, 87042 Limoges Cedex, France; ^6^ Limoges University Hospital, Department of Immunology, 87042 Limoges Cedex, France

**Keywords:** NX peptide, SCO-spondin, TSR, tumor angiogenesis, glioblastoma

## Abstract

Angiogenesis plays a critical role in glioblastoma growth and progression. We therefore aimed at evaluating the anti-angiogenic properties of an oligopeptide originating from SCO-spondin (NX) on a model of human glioblastoma. To this end, we studied the impact of NX treatment on human brain endothelial cells (HBMECs) alone or co-cultured with glioblastoma cells (U87-MG) on apoptosis, proliferation, migration and release of angiogenic factors. We further investigated the anti-angiogenic potential of NX on human glioblastoma cells grown on chorio-allantoic membrane (CAM) or in glioblastoma xenografts.

The results of our experiments showed that NX treatment impaired the microvascular network and induced a decrease in cell proliferation, vascularization and tumor growth in the CAM model as well as in xenotransplants. Interestingly, our *in vitro* experiments showed that NX impairs HBMECs migration but also regulates the release of angiogenic factors from U87-MG. These results are confirmed by the profiling of NX-treated U87-MG grown on CAM that highlighted modifications of several genes involved in angiogenesis.

In conclusion, NX inhibits tumorigenesis by impairing the ability of glioblastoma cells to induce angiogenesis and by inhibiting endothelial cell migration. This molecule might therefore be an interesting candidate for future cancer therapies.

## INTRODUCTION

Glioblastoma (GBM) is one of the most frequent and lethal forms of brain tumors. In spite of the advances in therapies, the survival of patients is generally inferior to 1 year [1]. The aggressiveness of GBM is associated with a rich and abnormal vasculature and treatments targeting angiogenesis represent a therapeutic strategy of choice to inhibit tumor proliferation and improve overall survival. Different anti-angiogenic approaches with variable efficiency were developed. Among these a number of peptide mimetics based on sequences derived from Thrombospondin-1 (TSP-1), an endogenous inhibitor of angiogenesis, have been tested [2]. Their anti-antigiogenic activity depends on subdomains of TSP-1 such as the thrombospondin type 1 repeat (TSR) as shown by the significant inhibition of *in vivo* tumor angiogenesis and growth in nude mice by single or multiple TSR motifs [3]. ABT-510, a TSP-1 mimetic drug, designed with TSRs as base, displayed anti-angiogenic properties in a phase 1 clinical trial in newly diagnosed glioblastoma [4, 5]. This peptide inhibited tumor growth *in vivo* and induced endothelial cells apoptosis *in vitro* [6]. Nevertheless, TSRs are not exclusively expressed in proteins belonging to the thrombospondin superfamily but also in proteins such as SCO-spondin, SEMA5A, 5B and F-Spondin [7]. SCO-spondin is a large glycoprotein involved in neurodevelopment which is strongly expressed in the subcommissural organ of mammals and contributes to the formation of Reissner’s fiber (RF) present in the central canal of the spinal cord. SCO-spondin carries 26 TSRs in human and is highly conserved in chordates, suggesting a key role of SCO-spondin's functions [8-11]. An original sequence consisting of twelve amino acids belonging to a TSR motif from SCO-spondin, the NX peptide, promotes cell-to-cell interactions and neurite outgrowth through an intracellular signal driven by β1 integrins independently of an RGD binding site in B104 cells [12]. NX possesses numerous properties in the central nervous system (CNS) such as neurite outgrowth, cell aggregation and differentiation, nevertheless its role in the vascular system is yet unknown [13, 14]. Axon guidance molecules isolated from development can play a dual role in nervous and vascular systems, like semaphorins which inhibit angiogenesis by blocking the interaction between VEGF and neuropilin-1 [15, 16]. Then, the similarities between SCO-spondin and these molecules led us to examine whether a peptide derived from the SCO-spondin might play an inhibitory role on tumor angiogenesis. Given that the original peptide is isolated from a protein of the CNS, we chose to assess its anti-angiogenic effect on Human Brain Microvascular Endothelial Cells (HBMECs) and to explore its potential anti-tumor activity in glioblastoma. Two different experimental models of glioblastoma have been carried out, *ex ovo* onto the chicken embryo’s chorio-allantoic membrane (CAM) and *in vivo*, with mouse xenografts. The comparison of the properties of NX with TSP-1 in tumor angiogenesis demonstrates that the NX affords a new type of anti-angiogenic agents, displaying distinct properties from TSP-1 and could be of interest for the development of therapeutic strategies to target highly vascularized tumors such as glioblastoma.

## RESULTS

### Anti-apoptotic and anti-migratory effects of NX on human brain microvascular endothelial cells

**Figure 1:**
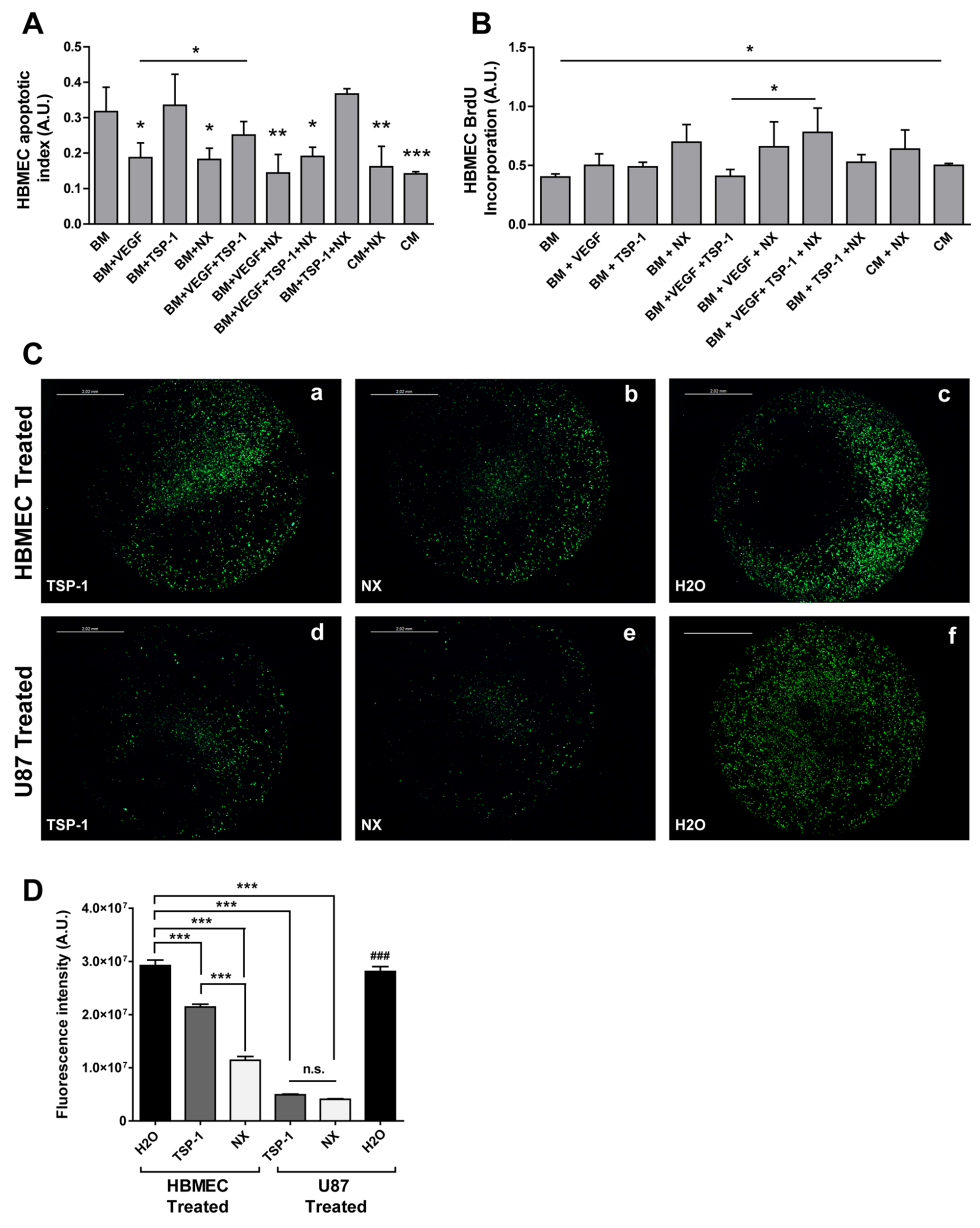
Functional effect of NX *in vitro* on proliferation, apoptosis and migration of HBMECs analyzed by **(A)** ELISA Cell Death assays, **(B)** BrdU Incorporation, **(C)** migration assay in Boyden chambers and **(D)** quantification of migration. A and B: Treatments by VEGF, TSP-1, NX were administered separately or in combination in basal medium (BM). Complete medium (CM) and BM were used as controls. P value<0.05 was considered significant. Comparisons between treatments were performed by using analysis of variances (ANOVA). * p<0.05; ** p<0.01; ***p<0.001 compared to BM. C: HBMECs were cultured in BM and seeded in the insert while U87-MG were seeded in the well with FBS free culture medium. HBMECs were treated with TSP-1 (a), NX (b) or H2O (control) (c). U87-MG were treated with TSP-1 (d), NX (e) or H2O (f). D: The number of migrating cells was quantified in 4 random images from each treatment group. Results are presented as mean +/- SEM from 3 independent experiments. Observations were performed using a fluorescent light microscope (magnification x20). Migrating and invading cells are reflected by fluorescent intensity. P value<0.05 was considered significant. *** p<0.001; ### p<0.001: U87-MG treated with H2O vs. U87-MG treated with TSP or NX.

Before evaluating the effect of NX on glioblastoma angiogenesis, the functions of NX were firstly studied on CNS endothelial cells, the HBMECs, to recapitulate the endothelial microenvironment of glioblastoma tumor. The NX properties were compared to those of TSP-1, used as anti-angiogenic control. As expected, an increased apoptosis rate was observed in HBMECs cultured in BM (Basal Medium) compared to CM (Complete Medium) (p=0.0007), the negative control, mainly due to the privation of essential growth factors for a prolonged period (Figure 1A). Similar apoptotic levels were observed in presence of TSP-1 treatment of HBMECs maintained in BM conditions. However, this basal apoptosis was significantly decreased following NX treatment (p=0.014), similarly to those obtained following VEGF treatment (p=0.02) a well-known angiogenic factor [17]. These data demonstrate that NX inhibits HBMECs apoptosis in BM culture conditions as well as VEGF. A treatment combining VEGF and NX impairs more significantly apoptosis (p<0.001). As expected, TSP-1 inhibits the anti-apoptotic effect of VEGF (p=0.0194). When VEGF and NX are combined with TSP-1, the apoptotic rate decreases compared to control (p=0.026). Our results confirmed the pro-apoptotic role of TSP-1 in presence of VEGF and showed an opposite effect of the NX peptide which prevents endothelial cell death. In U87-MG, NX and TSP-1 exhibited a similar effect decreasing apoptosis due to BM (BM vs. BM+NX, p<0.001; BM vs. BM+TSP, p<0.05). However, the action of NX is more significant than that of TSP-1 (Supplementary Figure 1A).

HBMECs proliferation rate at 24h significantly increased in CM (positive control) compared to BM (p=0.0367). Strikingly, the combination of NX with BM, VEGF and TSP-1 significantly increased proliferation compared to a similar condition without NX (p=0.039). No significant differences were detected in comparison with controls whatever treatment was applied (Figure 1B). In contrast to what was observed with HBMEC, adding NX to CM significantly decreased the proliferation rate of U87-MG (p=0.013) (Supplementary Figure 1B). Taken together these data suggest that NX inhibits the proliferation of U87-MG induced by CM without increasing apoptosis. Moreover, the peptide affects tumor cells and endothelial cells in a different way.

To further establish a functional role of NX in endothelial cell migration, we studied this mechanism in Boyden chambers. A significant decrease (p<0.001; Figure 1D) in migration from HBMECs compared to control (H_2_O; Figure 1Cc) was observed when they were directly treated with NX (Figure 1Cb). A similar inhibition of migration was exhibited by the treatment with TSP-1 alone (Figure 1Ca). In a second step, to determine whether NX could impair the endothelial migration induced by U87-MG, the treatments with TSP-1 (Figure 1Cd) or NX (Figure 1Ce) were directly applied on the glioblastoma cells. The migration of HBMECs was also significantly diminished (p=0.00028) as demonstrated by the quantifications (Figure 1D). This significant inhibition was also observed following the treatment of two distinct glioblastoma cell lines, A172 (p=0.011) and GL15 (p=0.0013) with NX (Supplementary Figure 2A).

Even though the NX peptide does not elicit apoptosis in endothelial cells, it inhibits the proliferation of tumor cells and can impair the migration of endothelial cells towards those, as demonstrated in U87-MG, A172 and GL15. Altogether these data confirmed that the inhibitory function of NX on brain endothelial cell migration could depend on an indirect action on tumor cells. Paracrine factors released by U87-MG cells could be affected by NX to modulate endothelial cell migration. To better understand the action of NX, we chose to analyze soluble factors released by U87-MG and HBMECs after NX-treated cells.

### NX treatment modifies the release of angiogenesis-related factors by endothelial and glioblastoma cells

**Figure 2:**
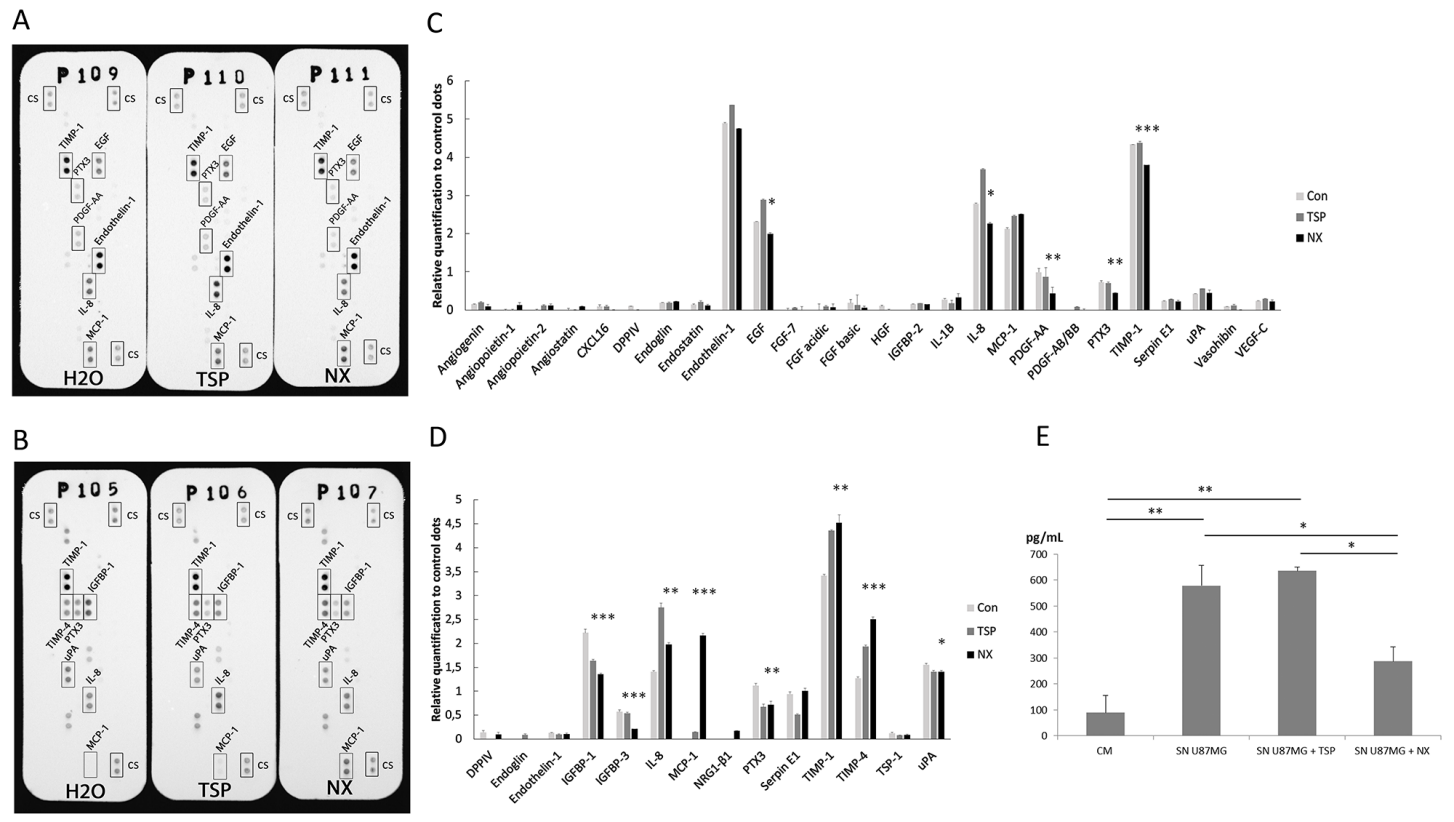
Analysis of angiogenic factors soluble or secreted by HBMECs and U87-MG HBMECs and U87-MG were treated with H2O (Con), TSP-1 or NX for 24h. Soluble factors from HBMECs supernatants (SN) were analyzed **(A)**; **(C)** and U87-MG **(B)**; **(D)** with proteome arrays. Relative quantifications of angiogenic factors released by HBMECs C) and by U87-MG (D) ;D) were normalized by control dots (cs). **(E)** The changes in VEGF-A release from U87-MG cells were revealed by ELISA and quantified by absorbance determination by spectrophotometry (405/650 nm). Comparisons between NX treatment and control were performed by using analysis of variances (ANOVA). * p<0.05; ** p<0.01 ***p<0.001.

Fifty-five soluble angiogenesis-related proteins were analyzed using a proteome array screening. Membranes were incubated with either supernatants from HBMECs or U87-MG cells (Figure 2A and 2B, respectively) in presence of NX or TSP-1 or not as control. Both TSP-1 and NX treatments, induced the expression of the similar angiogenesis-related factors from HBMECs compared to control (Figure 2A and 2C), however with lower levels of TIMP-1, PDGF-AA and IL-8 upon treatment with NX, and higher levels of IL-8, EGF and endothelin-1 following TSP-1 treatment.

By comparison to endothelial cells, NX or TSP-1 was then evaluated on U87-MG cells. Whereas IGFBP-1 secretion was significantly decreased in both cases compared to untreated cells, NX-induced levels were lower than those of TSP-1-induced (Figure 2B and 2D). An increase of TIMP-1, TIMP-4 and particularly MCP-1 (value ratio=2.1 *vs* control) levels were detected in U87-MG supernatants compared to untreated cells; NX-induced levels were higher to those obtained in presence of TSP-1, especially for MCP-1. Similarly, IL-8 secretion was significantly induced by TSP-1 (ratio: 2.90) compared to NX (ratio: 2) and controls (ratio: 1.38).

The pattern of expression of the secreted angiogenic factors following TSP-1 or NX treatment are distinct in U87-MG and HBMEC suggesting that NX activation was different in both cell types after treatment by TSP-1 or NX.

Interestingly, VEGF concentrations were similar in U87-MG and in U87-MG treated by TSP-1, whereas in NX-treated cells, VEGF level was significantly reduced (p=0.024) suggesting that NX inhibits the release of VEGF (Figure 2E). These observations were confirmed by the changes of VEGF concentrations in A172 and GL15 treated by NX (Supplementary Figure 2B). Indeed, VEGF levels were significantly decreased in A172 (p=0.037) and GL15 (p=0.035) after NX treatment compared to non-treated cells (SN A172, SN GL15). Similarly to previous results with U87-MG, VEGF concentration was significantly increased in TSP-1-treated cells compared to CM in both cell lines, A172 (p=0.025) and GL15 (p=0.002) (Supplementary Figure 2B).

Taken together these data assessed that NX modulates the migration of endothelial cells and the release of angiogenic factors, by both endothelial and glioblastoma cells, with different effects from those of the TSP-1. Therefore, to determine these functional properties, angiogenesis was evaluated on a chorio-allantoic membrane (CAM) model.

### NX inhibits the vascularization on a chorio-allantoic membrane model (CAM)

**Figure 3:**
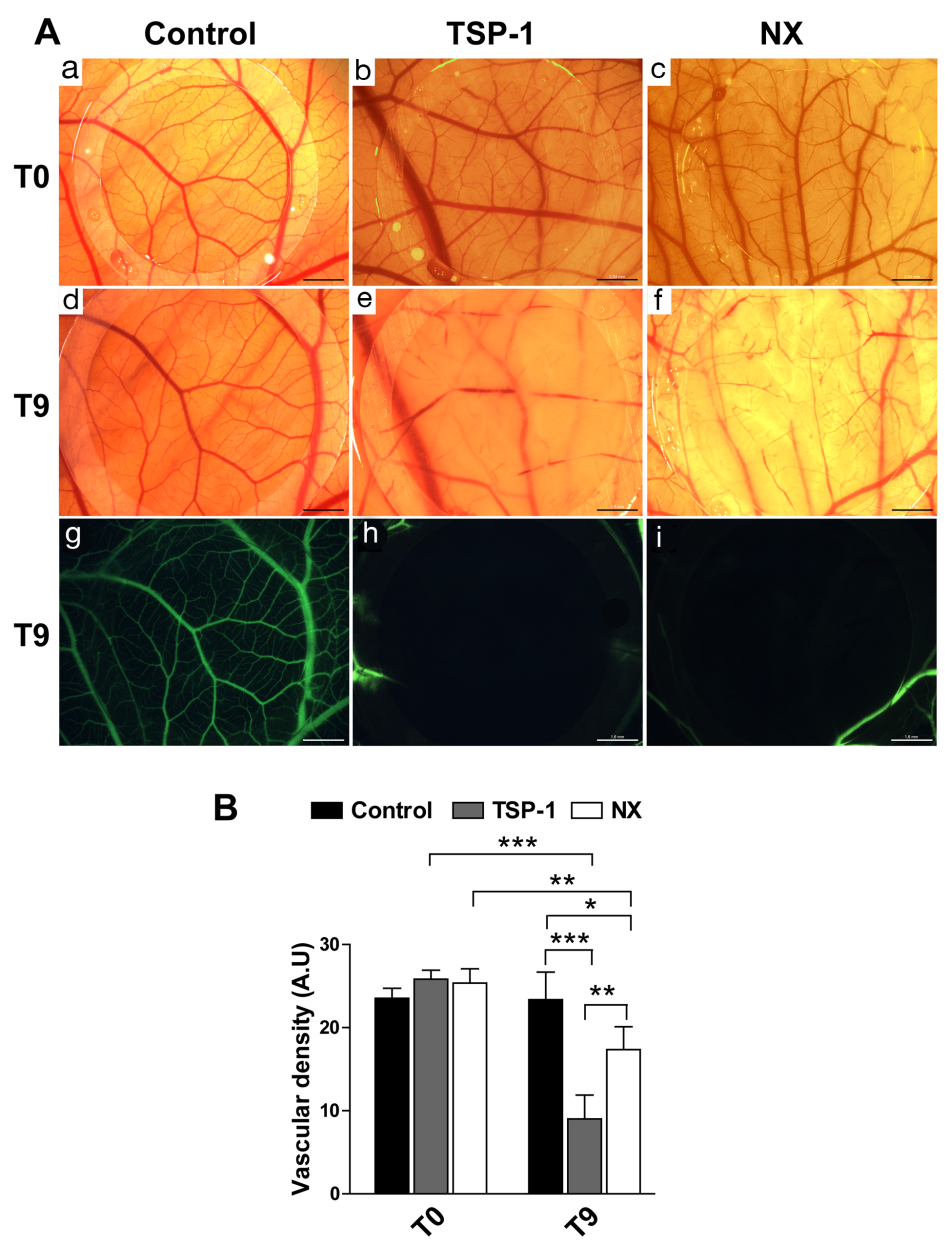
Vascularization of the CAM after treatment for 9h **(A)** a, b, c: CAM before treatments; d, e, f: CAM after treatments with H2O, TSP-1 and NX respectively; g, h, i: superficial vessels visualized with FITC-dextran after treatments with H2O, TSP-1 and NX respectively. Scale bars represent 2.04 mm for a, b, c and 1.6 mm for d, e, f, g, h, i. **(B)** The vascular density was quantified in 4 different images obtained in bright field. P value<0.05 was considered significant. Comparisons between treatments were performed by using analysis of variances (ANOVA). * p<0.05; ** p<0.01; *** p<0.001.

The inhibition of angiogenesis by NX has been challenged on the CAM model. The microvascularization of the CAM was significantly decreased after treatment for 9h (T9) with TSP-1 (Figure 3AE) or NX (Figure 3AF) compared to CAM at T0 (Figure 3AB, 3AC). The superficial micro-vessels were impaired and only the largest and deepest vessels were conserved following treatments with TSP-1 or NX (Figure 3AH and 3AI, respectively). The systemic injection of Dextran-fluorescein showed that this phenomenon did not appear on the control corresponding to the CAM treated with H_2_O (Figure 3AA, 3AD and 3AG). The quantification of vessel density on CAM confirms the macroscopic analyses (Figure 3B) (NX vs. control T0, p<0.001; NX vs. control T9, p=0.0102). These results confirmed that both NX and TSP-1 are putative inhibitors of angiogenesis in this CAM model. To determine their respective function in tumor growth, two experimental models of glioblastoma cell graft were then evaluated.

### NX prevents tumor angiogenesis on experimental glioma in CAM and induces changes in gene expression profile

**Figure 4:**
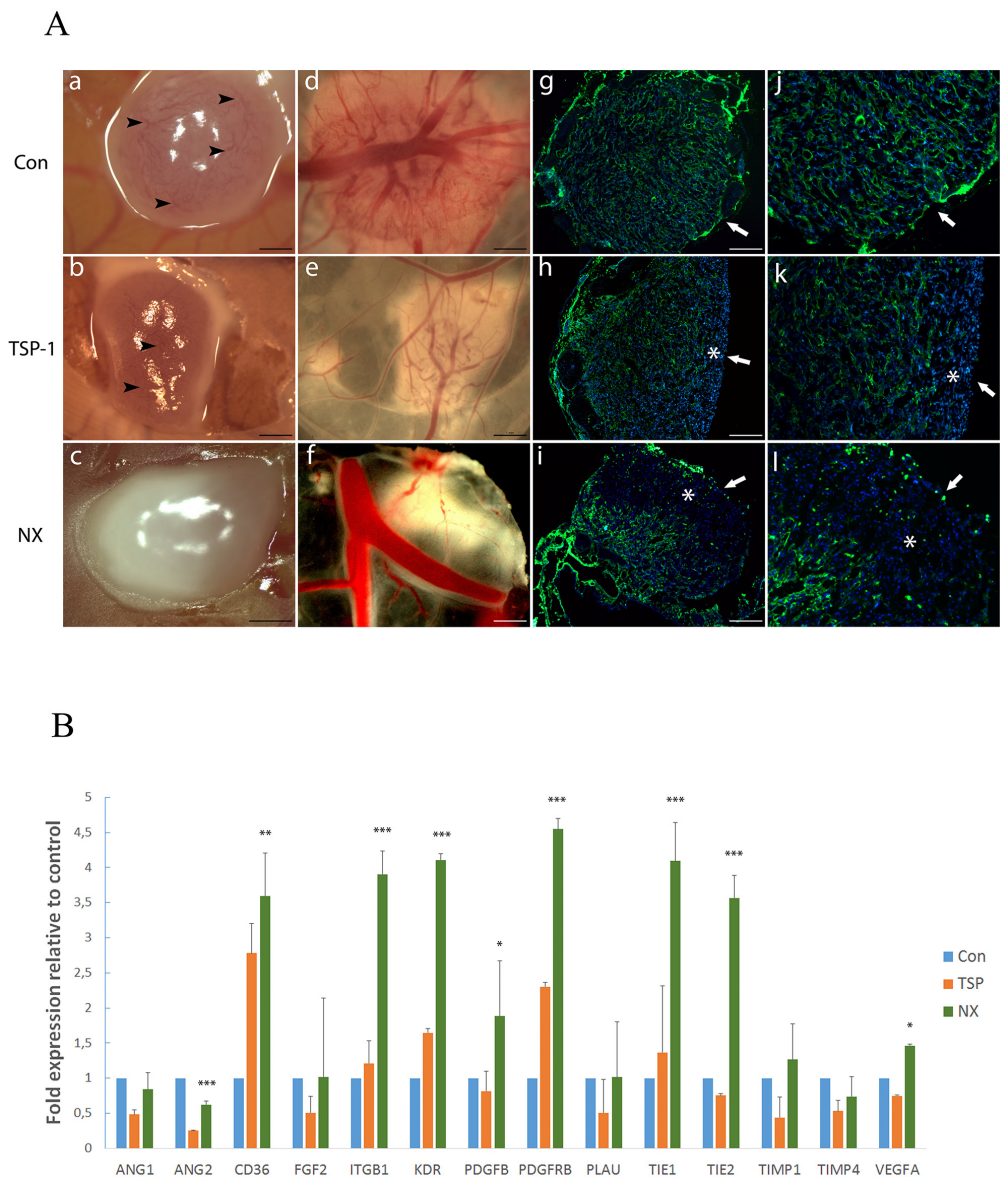
Analysis of tumor angiogenesis on the CAM model and angiogenesis implicated factors on tumors obtained **(A)**: Macroscopic aspect of tumors treated for 48h with H2O (a, d), TSP-1 (b, e) or NX (c, f), views from the top (a, b and c) and bottom (d, e and f) (scale bars 1 mm) and histological staining of vessels by SNA-lectin (green) and nuclei by DAPI (blue) of tumors treated with H2O (g and j), TSP-1 (h and k) or NX (i and l) (g, h and i: scale bars represent 1 mm; j, k and l: magnifications x2 from g, h and i, respectively). Arrowheads correspond to visible vessels; arrows correspond to the surface treated and stars to areas without vessels. **(B)**: Quantitative analysis of mRNA in tumors grown on the CAM. The different genes are represented by a relative expression compared to control tumors treated with H2O. P value<0.05 was considered significant. * p<0.05; ** p<0.01; *** p<0.001.

U87-MG cells were grafted on the CAM and a glioblastoma tumor was observed at 72 hours. A macroscopic observation showed differences in control and treated tumors: the control tumors were translucent with numerous apparent, tortuous and dilated blood vessels (black arrow head) at the tumor surface providing evidence for functional tumor capillaries (Figure 4A,a) and larger vessels penetrating underneath were observed (Figure 4A,d). By contrast, tumors treated with NX were lactescent with white necrotic foci, and no apparent vessel on the top of the tumors (Figure 4A,c) without vessels penetrating into the tumor (Figure 4A,f). By comparison to NX conditions, tumors treated with TSP-1 only displayed a translucent ring around the tumor and lesser vessels on the top (Figure 4A,b; black arrow head) and the back of the tumor (Figure 4A,e) compared to controls.

These different aspects of the tumors were confirmed by the staining of chick vessels using the SNA-1 lectin. Indeed, in contact with NX, the vascular density was significantly reduced in the treated area of the tumors (asterisk, Figure 4A,i and 4A,l) compared to controls (Figure 4A,g and 4A,j) (Supplementary Figure 3; p<0.001). Similar observations were detected in TSP-1-treated tumors (Supplementary Figure 3; p<0.0019). Nevertheless, the vascularization was significantly more decreased in NX condition than in TSP-1- treated tumors (asterisk, Figure 4A,h and 4A,k; p=0.0011, Supplementary Figure 3). The quantification of vessel staining and density supports that topical treatment (white arrow) of experimental glioblastoma with NX impaired vascularization. To explain the changes observed in these tumors, we chose to look at the events occurring at the mRNA level.

Several genes (14) were chosen to determine whether transcriptomic changes induced by TSP-1 and NX treatments occur. The choice of studied genes was driven by their involvement in main angiogenesis mechanisms and their relationship with others proteins known to interact with SCO-spondin or NX. The comparative analysis performed with 14 genes revealed that 9 genes are significantly deregulated upon NX treatment compared to control (Figure 4B). We distinguished 3 gene expression patterns of NX and TSP-1 compared to control: (i) 5 overexpressed genes upon TSP-1 or NX treatment (*CD36, ITGB1, KDR, PDGFRB, TIE1*); (ii) 1 downregulated gene upon TSP-1 or NX treatment (*ANG2*); (iii) 3 genes displayed a differential expression between NX and TSP-1 (*PDGFB, TIE 2, VEGFA*). No significant differences of expression were observed in 5 genes. Overall, 9 genes shared similar expression levels upon both treatments, suggesting a similar anti-angiogenic activity of the molecules, which could be dependent of distinct mechanisms of action as shown by the specific overexpression of *ITGB1* and *KDR* induced by NX.

### Proliferation rate of experimental tumor grafted on the CAM is significantly decreased by NX

**Figure 5:**
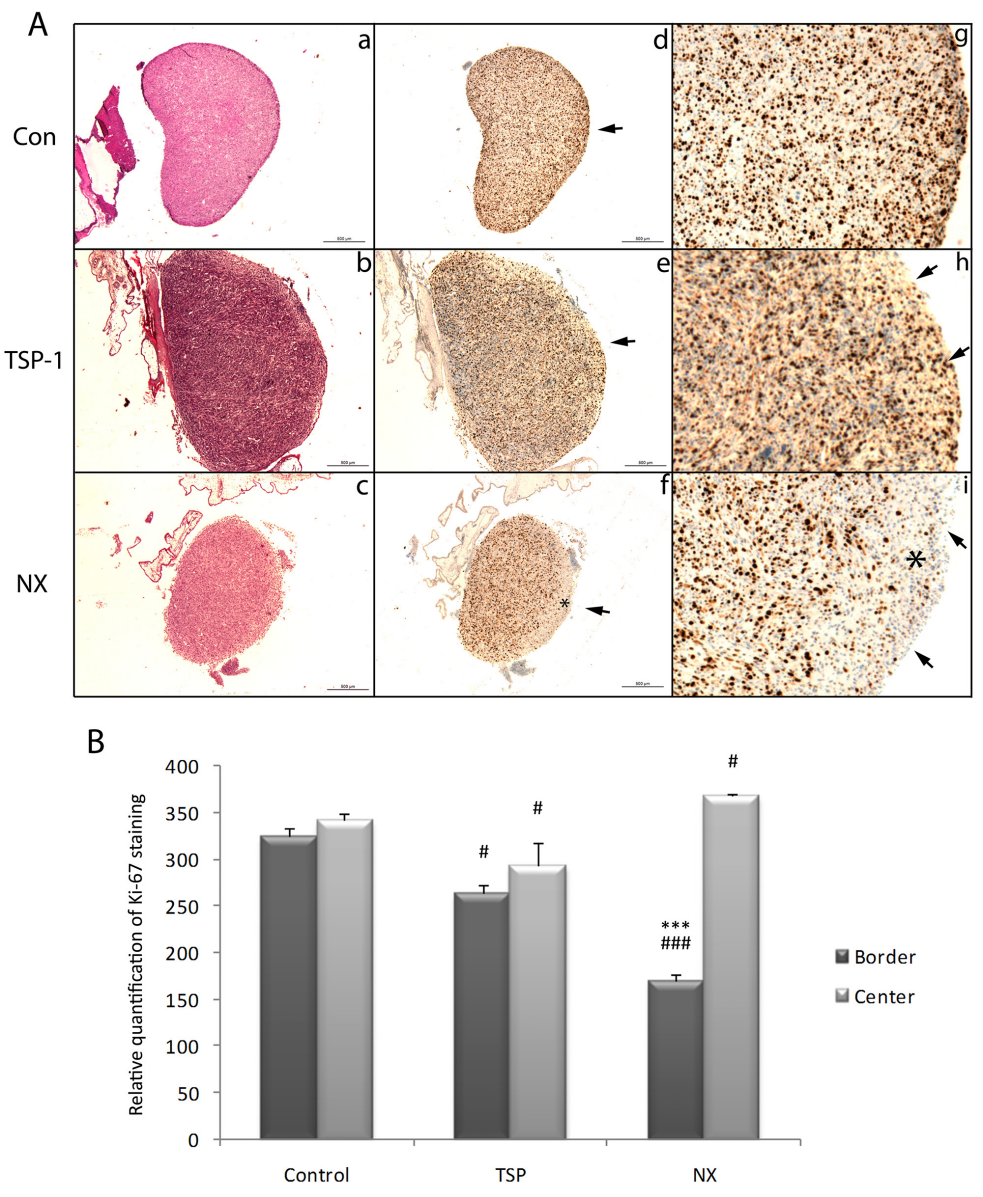
Histological and immunohistological analysis of proliferation in tumors from the CAM after treatments Two treatments with either H2O, 250 µg/mL NX or 10 µg/mL TSP-1 were administered in 48h. Another treatment was administered 24h later. A, B and C: Hematoxylin Eosin Safran stainings of tumors treated with H2O, TSP-1 or NX respectively. D, E and F: Ki-67 immunostaining of tumors treated with H2O, TSP-1 or NX respectively (scale bars 500 µm). G, H and I: magnifications (x2) from D, E and F borders, respectively. Arrows correspond to the surface treated and stars to areas without Ki-67 staining. Ki-67 staining was quantified using Image J and comparisons between treatments and control and between tumor center and border were performed by using analysis of variances (ANOVA test). P value<0.05 was considered significant. * p<0.05; ** p<0.01; *** p<0.001 compared to center of the same tumors; # p<0.05; ## p<0.01; ### p<0.001 compared to control.

The tumors obtained on CAM shared homologies with human glioblastoma morphologically and structurally. HES staining confirmed hypercellular pleomorphism tumor with mitotic figures, multinucleated tumor cells, bizarre nuclei and large cell heterogeneity (data not shown). Similar markers of human glioblastoma cells were also expressed as confirmed by comparable expression of specific proteins of human glioblastoma such as vimentin and nestin (data not shown). However the HES staining did not show any particular differences between control tumors (Figure 5A,a) and TSP-1- or NX-treated tumors (Figure 5A,b and 5A,c respectively). The histology was also similar to those of high grade astrocytoma. It corresponds to a hypercellular pleomorphism tumor with mitotic figures, multinucleated tumor cells, bizarre nuclei. Tumors grafted on CAM also displayed a large cell heterogeneity with either coagulation necrosis or microvascular proliferation areas. Nevertheless, the proliferation rate evaluated by Ki-67 positive cells was drastically decreased in the tumor areas exposed to NX (bold asterisk, Figure 5A,f and i) compared to control or TSP-1 (p=0.0004; Figure 5A,d, 5A,g and 5A,e, 5A,h and 5B). The staining quantification confirms that the proliferation rate was significantly decreased at the periphery of the tumor in which the treatment was deposited (p<0.0001; Figure 5B) compared to the center (Figure 5A,f, A,i). These areas in which the proliferation is diminished are correlated with the areas devoid of micro-vessels as assessed by SNA staining. This phenomenon was less important after treatment with TSP-1 (Figure 5A,e and 5A,h) and not detected in control tumors (Figure 5A,d and 5A,g). Our *in vivo* results are consistent with *in vitro* observations in which the proliferation of U87-MG was inhibited by NX treatment.

### NX prevents tumor growth by inhibiting cell proliferation and vascularization *in vivo*

**Figure 6:**
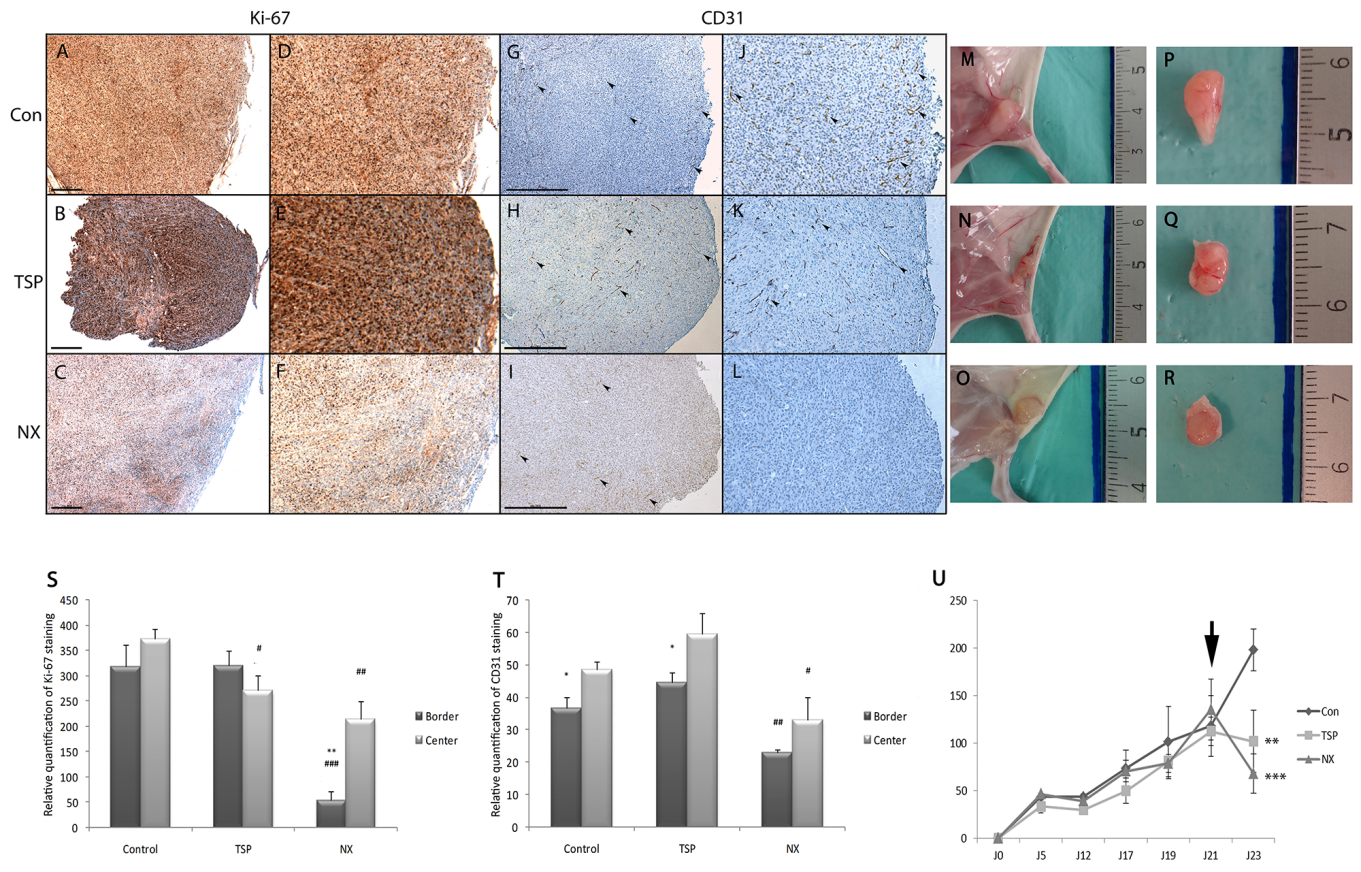
NX activity on proliferation, vascularization and tumor growth in mice Treatments were administered by diluting the compounds in 100 µL hydrogel (CosiGel, Interchim) at a concentration of 250µg/mL for NX and 10µg/mL for TSP-1 before injecting at the periphery of the tumors. 9 animals for each group were used. **(A)**, **(B)** and **(C)**: Ki-67 immunostaining of tumors treated with H2O, TSP-1 or NX respectively. **(G)**, **(H)** and **(I)** CD31 immunostaining (arrowheads) of tumors treated with H2O, TSP-1 or NX respectively (scale bars 500 µm). **(D)**, **(E)** and **(F)** are magnifications (x2) from A, B and C; **(J)**, **(K)** and **(L)** are magnifications (x2) from G, H and I respectively. **(S)** and **(T)** Ki-67 and CD31 stainings were quantified using Image J. Comparisons between treatments and control and between tumor center and border were performed by using analysis of variances (ANOVA test). P value<0.05 is considered significant. * p<0.05 compared to center of the same tumors; # p<0.05; ## p<0.01 compared to control. **(M)** and **(P)** tumor from mice after hydrogel treatment alone. **(N)** and **(Q)** tumor from mice after TSP treatment. **(O)** and **(R)** tumor from mice after NX treatment. **(U)** Tumor growth measures 5, 12, 17, 19, 21 and 23 days after cell injections. Treatment (hydrogel alone, TSP and NX) injections were realized at day 21 (black arrow). Tumor size in comparison to control’s was performed by using analysis of variances (ANOVA test). ** p<0.01; *** p<0.001.

To evaluate the properties of NX in the growth of glioblastoma, we used a model of subcutaneous transplantation of tumor cells in nude mice. No differences were observed in cell proliferation (Ki-67 positive cells) in tumors treated with TSP-1 (Figure 6B and 6E) and controls (Figure 6A and 6D). In contrast, the proliferation was decreased in peripheral areas in NX-treated tumors (Figure 6C and 6F) (p<0.0001; Figure 6S). A similar staining pattern was observed with the endothelial marker CD31 (arrows) showing a decreased staining in peripheral areas in tumors treated with NX (Figure 6I and 6L) (p=0.008, Figure 6T), while this labeling was homogenous in controls (Figure 6G and 6J) and TSP-1-treated tumors (Figure 6H and 6K). Furthermore, the tumor volume was significantly decreased at day 23 with NX treatment compared to the control (p<0.0001; Figure 6U). Tumor volume is significantly reduced by NX treatment compared to TSP-1 (p=0.0015; Figure 6U). These results suggest an inhibitory effect of NX both on tumor and endothelial cells, reducing neovascularization and tumor growth *in vivo*.

## DISCUSSION

The present report shows for the first time that a short oligopeptide, NX, derived from a large protein secreted in the CNS, the SCO-spondin, could inhibit angiogenesis *ex ovo* and *in vivo*, in a glioblastoma model. This effect could be mediated by interactions of this molecule with tumor cells or the tumor microenvironment. Furthermore, we demonstrate that NX induces a tumor angiogenesis inhibition superior to TSP-1 effect, without apoptosis induction of endothelial cells *in vitro*.

Moreover, when endothelial cells are co-cultured with U87-MG cells in a Boyden chamber, NX inhibits their migration. This indirect effect of NX on angiogenesis is not restricted to U87-MG but it is observed also in two distinct glioblastoma cell lines. Therefore, we decided to investigate whether this effect relied on the control of glioblastoma cells’ secretome. We then analyzed factors released by U87-MG and HBMECs. *In vitro*, the different treatments do not significantly affect their release by endothelial cells. In contrast, the released factors from U87-MG cells were different depending on their treatment. Indeed, NX treatment reduced the secretion of VEGF from three different glioblastoma cell lines, which could be related to the decrease of endothelial cell migration. Furthermore, the down-regulation of several proangiogenic factors (uPA, PTX-3, and IGFBP-1) known to foster tumor angiogenesis suggests that NX might indirectly prevent angiogenesis by affecting the tumor microenvironment [18-20]. The dowregulation of uPA upon NX treatment could explain the increase of TIMP-1 release by U87-MG cells as previously reported *in vitro* and *in vivo*. Moreover, uPA blocking by NX could reduce VEGF available for angiogenesis through secretion of SVEGFR1 by glioblastoma cells [18]. Similarly, the up-regulation of both anti-angiogenic factors, TIMP-4 and IGFBP3, suggests that NX treatment prevent tumor angiogenesis by controlling the secretome of U87-MG cells [21-23]. Only 2 proteins (MCP-1 and IL-8) considered as proangiogenic factors are up-regulated in the supernatant from U87-MG upon NX treatment [24-26]. However, in human malignant gliomas, the level of MCP-1 mRNA and protein expression is correlated with the macrophage infiltration and immunosuppression but not with angiogenesis [27]. Concerning IL-8, its secretion was not blocked by NX in GBM cells. Nevertheless, in HBMEC, NX treatment confirms its anti-angiogenic effect by significantly inhibiting IL-8 release, but also both pro-angiogenic factors, TIMP-1 and EGF.

The changes in expression level of angiogenesis related proteins support a regulatory effect of NX in angiogenesis. However, the regulatory interactions between these proteins cannot be fully assessed *in vitro*, and require to be studied in a model allowing interactions with critical receptors and the microenvironment.

The chick CAM model allows to study the relationship between microenvironment and endothelial cells and permits to assess the anti-angiogenic properties of NX [28]. Only the large vessels conserved their morphology while small vessels were impaired by the treatment. The significant decrease of the microvascularization in the treated area with TSP-1, a reference molecule to analyze anti-angiogenic properties and NX suggests a similar anti-angiogenic effect of both molecules in this model.

To confirm *in vivo* the results obtained in CAM model, NX as well as TSP-1 were tested on an experimental glioblastoma model on the CAM. Macroscopic observations showed alterations in the color and size of the tumors following NX treatment. Indeed, a white aspect appears on the surface of NX treated tumors suggesting the formation of necrotic areas similar to those previously described with other anti-angiogenic molecules [29]. TSP-1 and NX seem to induce similar effects on tumor vascularization, but SNA-1 staining confirmed that the vascular network was markedly more altered by NX than by TSP-1 treatment suggesting that NX is a most efficient anti-angiogenic factor. The decrease of the proliferation rate in the same areas correlated with the inhibition of the local vascularization.

Given the structural similarities between TSP-1 and NX, we chose to analyze the expression levels of genes involved in TSP-1 inhibition mechanisms [7]. CD36 and β1 integrin are considered the main receptors for TSP-1 mediating anti-angiogenic properties through the formation of complexes including VEGFR-2 [30, 31]. Strikingly, β1 integrin mRNA was not overexpressed after treatment with TSP-1, while the mRNA level of each gene related to the complex is increased following NX treatment. These observations support that NX might promote the expression of specific genes and enhance this inhibitory association. The mechanism of action of NX and TSP-1 might be different although they share structural homologies [7]. Involvement of β1 integrin in NX activity has been already reported and do not require RGD motifs recognizing [12]. This mechanism of action can also distinguish NX from ABT-510, which is a TSP-1 mimetic isolated from a different part of the TSR domain and which induces a decrease in expression of VEGF and VEGFR-2 *in vitro* [32]. Several genes (*PDGF-B, PDGFRβ, TEK, TIE-1)* encoding for proteins implicated in vessel stabilization and maturation are overexpressed following NX treatment. The resulting proteins are targeted by several angiogenesis inhibitors such as Imatinib which blocks PDGFRβ, implicated in vessel stabilization [33, 34]. However, its concomitant expression with VEGF induces the formation of receptor complexes consisting of PDGFRβ and VEGFR2 which prevent angiogenesis events and leads to a reduction in vascular pericyte coverage compared to tissues treated with PDGF alone [35]. Furthermore, the strong upregulation of these mRNA, as well as those corresponding to *ITGB1* and *KDR (coding for* VEGF-R2) was only observed when NX was applied, thus pointing out a new anti-angiogenic mechanism which was confirmed *in vivo*.

Experiments in mice confirmed the inhibitory function of NX in angiogenesis demonstrated previously on the CAM model, with the same duration of treatment (48h). A superficial decrease of vascularization and cell proliferation was observed due to the local administration of NX which results in a tumor volume reduction. The effect of TSP-1 seems weaker compared to NX. Although TSP-1 has been reported to have potent anti-angiogenic and anti-tumorigenic effects, its large size and complex functions limit its possibilities of use as a therapeutic antitumor strategy in humans. In order to stabilize the release of NX and compare our results with tumors growth on the CAM, treatments were achieved using a hydrogel matrix delivering the molecules close to the tumors by resorbing. The anti-angiogenic effects of NX do not seem to depend on direct apoptosis of endothelial cells or vascular normalization. Such properties are distinct from ABT-510 which induces cell death in Microvascular Endothelial Cells (MvECs), promotes vascular remodeling and inhibits tumor growth [6, 32, 36, 37]. However ABT-510 displays a different sequence and could depend on another receptor than NX.

In conclusion, NX seems to possess different properties compared to TSP-1 as it does not directly induce apoptosis on brain microvascular endothelial cells *in vitro* but supports their survival. However, *in vivo*, NX reduces the vascular density and inhibits tumor cell proliferation as reported with other molecules like Sunitinib or Sorafenib [29, 38, 39]. NX was not specifically directed against a specific tumor target but could affect both the microenvironment and tumor cells. As it might cumulate both effects on angiogenesis and tumorigenesis as described in this study, its mechanisms differ from the other angiogenesis inhibitor. Indeed, its effects include an upregulation of the transcripts of several key genes in anti-angiogenic signaling pathways which might play a crucial role in the formation of an inhibitory complex associating CD36, integrin β1 and VEGFR-2. These findings point out the interest of NX in tumor angiogenesis, which might provide a new anti-angiogenic molecule for the development of promising therapeutic strategies.

## MATERIALS AND METHODS

### NX peptide

NX peptide was obtained by chemical synthesis (Polypeptide Laboratories). The sequence has been previously detailed [9]. For each experiment, NX was extemporaneously resuspended in sterile water at 250 μg/mL.

### Cell lines and cell culture

U87-MG, GL15 and A172 cell lines were purchased from the American Type Culture Collection (ATCC/LGC Promochem). Cells were grown in Dulbecco’s modified Eagle’s medium (DMEM) (Invitrogen) supplemented with 10% FBS (Invitrogen), 100 IU/ml penicillin G, 100 μg/ml streptomycin, 2 mm glutamine. HBMECs (ScienCell) were cultured in EGM2-MV medium (Lonza), later referred to as complete medium (CM). HBMECs were used between passages 5 and 8.

### *In vitro* treatments

10 μg/mL recombinant human thrombospondin-1 (R&D Systems) or 250 μg/mL NX were diluted in complete culture medium (CM), or basal medium (BM) consisting in EBM2 (Lonza) with 2% FBS. TSP-1 contains 3 TSR motifs while NX corresponds to a consensus sequence deduced from one of the TSR motifs of the SCO-spondin. Thus, the molar concentration of NX is higher than TSP-1 in all the experiments. The time for treatment was 24h. These conditions were applied to all *in vitro* experiments.

### *In vitro* proliferation and cell death assays

HBMEC and U87 proliferation was assessed by BrdU incorporation after a 24h treatment with Proliferation assay kit according to manufacturer’s recommendations (Cell Signaling, Ozyme). Cell death was analyzed by determination of cytoplasmic histone-associated-DNA-fragments with ELISA Cell Death Plus (Roche Applied Science) according to manufacturer’s recommendations.

### Endothelial migration assay

After growth factors starvation in BM for 12h, 2.5 x 10^4^ HBMECs were seeded in a transwell insert (Endothelial Angiogenesis Array, BD Biosciences) incubated with BM supplemented with 2% FBS alone or with 250 μg/mL NX or 10 μg/mL TSP-1. To determine whether NX could inhibit the endothelial migration induced by U87-MG, A172 or GL15, 10 x 10^3^ cells were seeded in the bottom well in culture medium and treated with NX in comparison with TSP-1. The system was incubated for 20h then cells were stained with calcein AM (BD Biosciences) and membranes were analyzed using a binocular light microscope (MZFL3, Leica).

### Proteome analysis

Cells (U87-MG or HBMECs) were seeded in 6-well plates and treatments were administered during 24h. Supernatants were collected and 55 angiogenesis-related proteins were studied using Proteome Profiler Angiogenesis Array (R&D Systems) according to manufacturer’s recommendations. The levels of the angiogenesis-related proteins were expressed as a ratio relative to the mean of the positive controls. Only variations of ratio superior to 0.5 were considered significant.

### Measurements of protein levels of vascular endothelial growth factor (VEGF) by ELISA

U87-MG, A172 or GL15 cells were cultured in 6-well plates and treatments (TSP-1 or NX) were administered during 24h. The total proteins from supernatants were quantified using Human VEGF Standard Development Kit (Peprotech) according to the manufacturer’s protocol. The protein concentrations of VEGF were normalized and expressed as pg/mL of supernatant.

### Evaluation of anti-angiogenic effects on an experimental glioblastoma in the chick CAM

Fertilized chicken eggs (*Gallus gallus*) (HAAS, Kaltenhouse) were incubated at 38°C and 65% humidified atmosphere. For the CAM assay, on ED9, a silicon ring was placed on the CAM and 30 μL of treatments (H_2_O, TSP-1 or NX) were deposited onto the center of the ring. After 9h, a systemic injection of fluorescein-dextran (Sigma) was performed. The vascular network was visualized using MZFL3 fluorescent stereomicroscope (Leica). The quantification of vascular density at T0 and T9 in the different conditions were carried out in bright field images with Image J.

For experimental glioma assay, at ED8, 2.5 x 10^6^ cells in 20 μL of cell suspension were deposited after gentle scratching of the surface. At ED11, a first treatment with either H_2_O, TSP-1 or NX was deposited onto the surface of the tumors. Another treatment was administered 24h later. At ED14, the experimental gliomas were observed and pictures were taken under a MZFL3 stereomicroscope (Leica). The tumors were recovered and fixed with 4% paraformaldehyde for achieving immunohistochemistry analysis, while for mRNA studies, tumor was stored at -80°C.

### Evaluation of anti-angiogenic effects *in vivo*

Nude mice (NMRI, Janvier) were injected 2.5 x 10^6^ U87-MG cells suspended in 30 μL PBS and tumor growth was monitored during 23 days. Local treatments of the tumor were administered by diluting the compounds in 100 μL hydrogel (CosiGel, Interchim) at a concentration of 250μg/mL for NX and 10μg/mL for TSP-1. Treatments were administered at day 21 after cell injection and animals were sacrificed at day 23. Measurements of the tumors were achieved after total resorption of the hydrogel using the following equation V= 0.5 x (L x l2). The protocols were carried out according to the guidelines for the care and use of laboratory animals and were approved by the animal Care and Use Committee.

### Histology and immunohistochemistry

Experimental gliomas from CAM and mouse/mice tumors were fixed with formol 10% and embedded in paraffin (Pathology department, CHU Limoges). Tumors were cut into 4 μm sections and stained with hematoxylin-erythrosin-safran. To characterize mouse/mice vessels, an immunohistochemistry with CD31 (1/30, Histonova) was performed. Ki-67 (1/50, Dako) immunostaining was processed with automated immunohistochemistry using the BenchMark technology (Ventana medical Systems). The sections were observed using a DMRX microscope (Leica). Negative controls were obtained by using an isotypic control, mouse IgG (Santa Cruz Biotechnology), or by omitting the primary antibody.

### SNA lectin staining

Chick blood vessels were visualized by using FITC-coupled SNA-1 (Sambuccus Nigra agglutinin) lectin (1:200, Vector Laboratories). Tumors were fixed in PBS-paraformaldehyde 4 % and embedded in optimal cutting temperature medium (Tissue Tek, Sakura). Frozen sections (8 μm) were stained with SNA-1 coupled FITC at a 1/200 dilution in TBS-Tween20 0.1 % pH 8.4 for 1h at room temperature. Nuclei were visualized with DAPI (Invitrogen) and fluorescent labelling was viewed by DMRX fluorescent microscope (Leica). SNA staining were quantified by using image J.

### Total RNA purification and reverse transcription

Total RNA were extracted and purified from tumors grown on CAM with RNeasy (Qiagen) as described by manufacturer. RNA integrity was assessed on an Agilent 2100 bioanalyser using the RNA 6000 Labchip kit (Agilent Technologies). cDNA were generated using a High-Capacity cDNA Reverse Transcription kit (Applied Biosystems) as described by the manufacturer from 1.5 μg of total RNA.

### Quantitative real-time PCR

The expression levels of genes (primers and probes table in supplementary materials) were analyzed by real-time PCR using TaqMan gene Expression Master Mix (Applied Biosystems) on the StepOne plus Real time PCR system (Life technologies). Samples were analyzed in triplicates, and the data were normalized using the HPRT gene as an internal control. Relative quantification (RQ) of the genes in a sample was determined according to the equation 2^- DDct^ [40].

### Statistical analysis

Data were expressed as mean ± SD. Comparisons between groups were performed by ANOVA followed by Tukey’s multiple post-test analysis. Statistical analysis was performed <0.05 was considered significant.

## SUPPLEMENTARY MATERIALS FIGURES


