# Supplementary material for: SCO-spondin oligopeptide inhibits angiogenesis in glioblastoma
Source: Oncotarget. 2017 Sep 12;8(49):85969–83. doi: 10.18632/oncotarget.20837 (PMC5689660; doi:10.18632/oncotarget.20837)
Supplement: Supplementary file 1 [file oncotarget-08-85969-s001.pdf]

SCO-spondin oligopeptide inhibits angiogenesis in glioblastoma

SUPPLEMENTARY MATERIALS

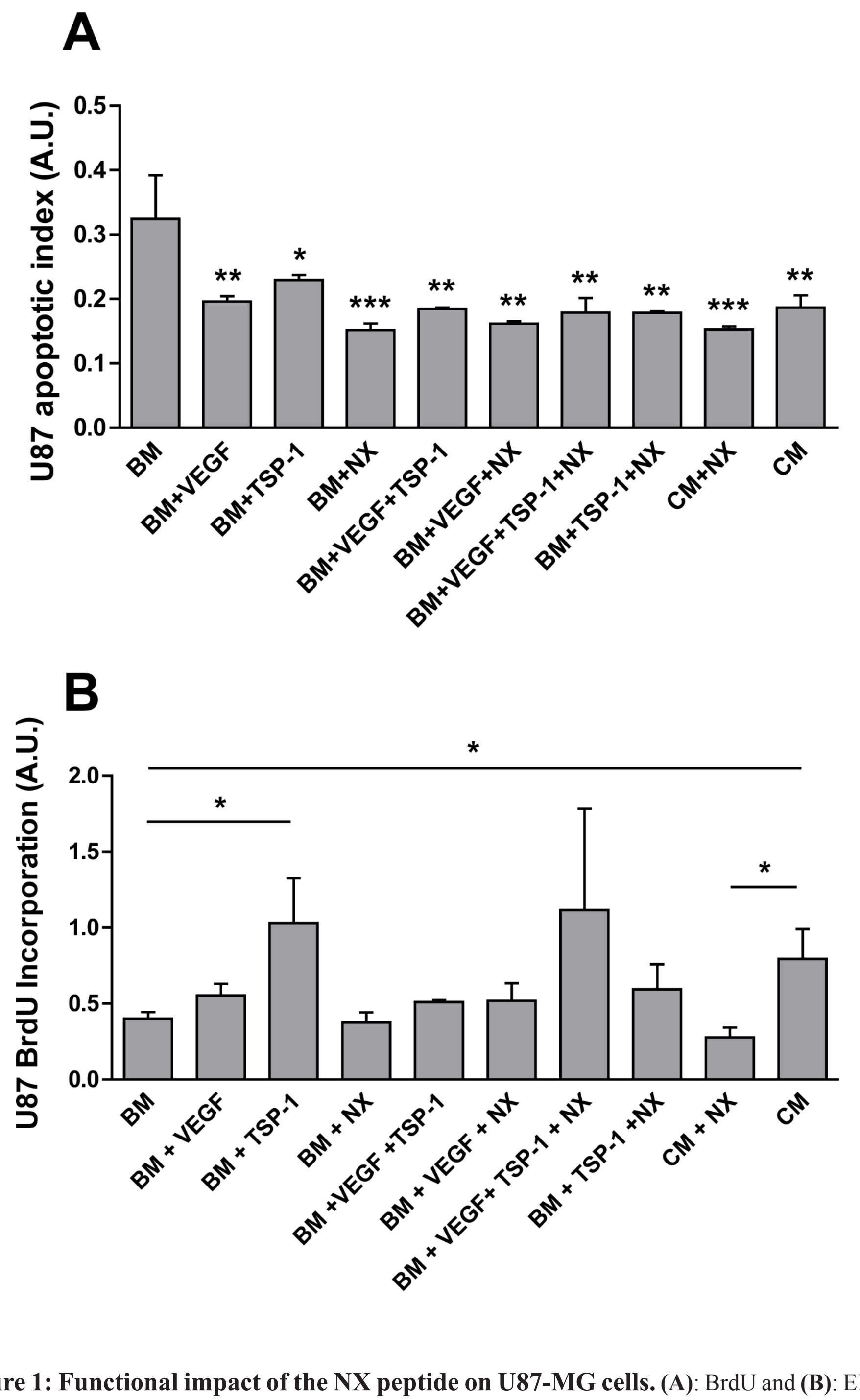

Supplementary Figure 1: Functional impact of the NX peptide on U87-MG cells. **(A)**: BrdU and **(B)**: ELISA cell death assays. Treatments by VEGF, TSP-1 and NX were administered separately or in combination in basal medium (BM). Complete medium (CM) and BM were used as controls. P value<0.05 was considered significant. Comparisons between treatments and control (BM) were performed by using analysis of variances (ANOVA). * p<0.05; ** p<0.01; *** p<0.001.

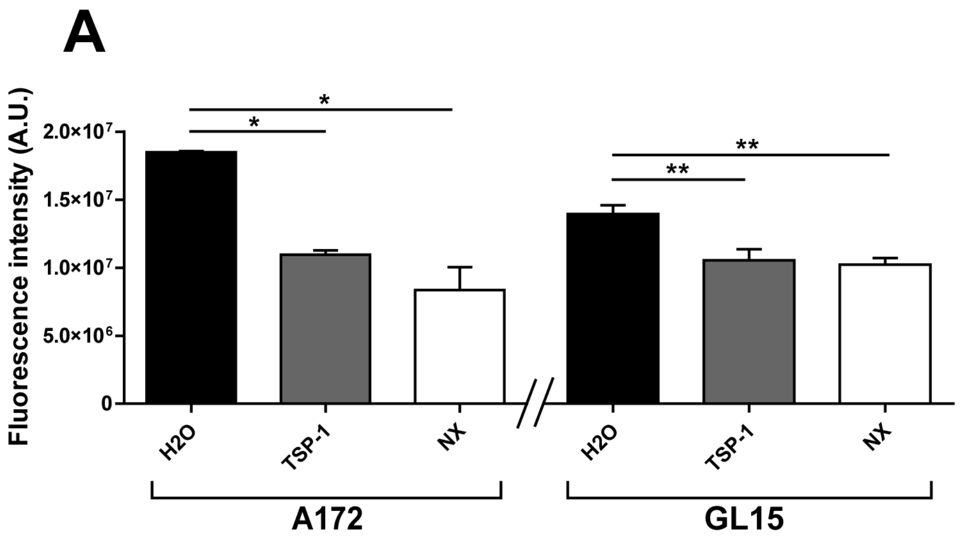

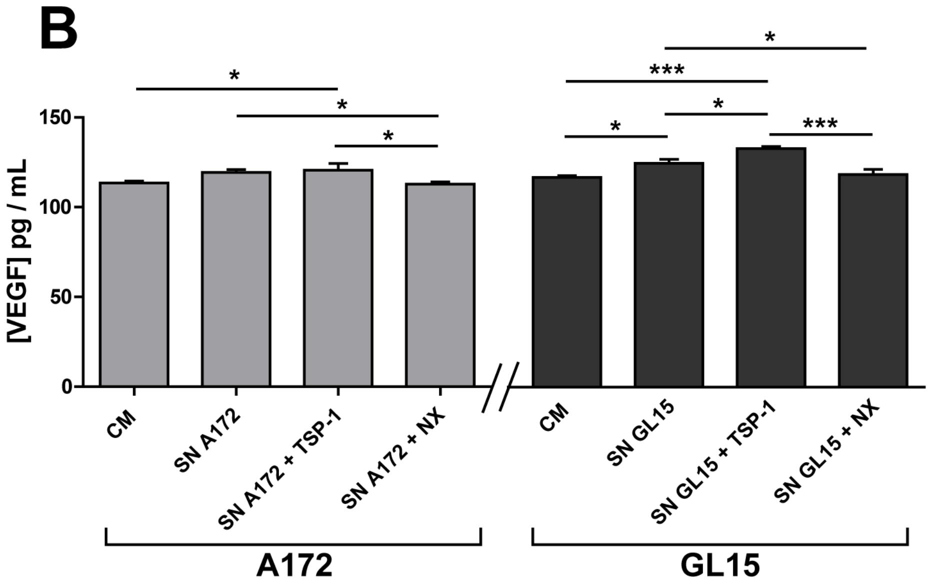

Supplementary Figure 2: Effect of NX and TSP-1: (A) on migration of endothelial cells following treatment of two distinct glioblastoma cells (A172 and GL15); (B) on VEGF released by A172 and GL15 cells. (A) Migration assay in boyden chamber with HBMECs seeded in the insert and in the well, A172 or GL15 in culture medium. A172 or GL15 were treated with TSP-1, NX or H2O (control). The number of migrating cells was quantified in 4 random images from each treatment group. Results are presented as mean {plus minus} SEM from 3 independent experiments. Observations were performed using a fluorescent light microscope (magnification x20). The capacity of treated glioblastoma cells to induce HBMEC migration is closely linked to the number of migrating cells reflected by fluorescent intensity. **(B)** VEGF concentration, corresponding to VEGF released by NX or TSP-1 treated A172 or GL15 cells, was quantified by ELISA and absorbance determination by a spectrophotometer (405/650 nm). P value<0.05 was considered significant. In both glioblastoma cell lines, NX treatment significantly decreased VEGF released by cells compared to non-treated cells (SNA172 and GL15). Comparisons between the treatments and controls were performed by using analysis of variances (ANOVA). * p<0.05; ** p<0.01 ***p<0.001. (SN: Supernatant)

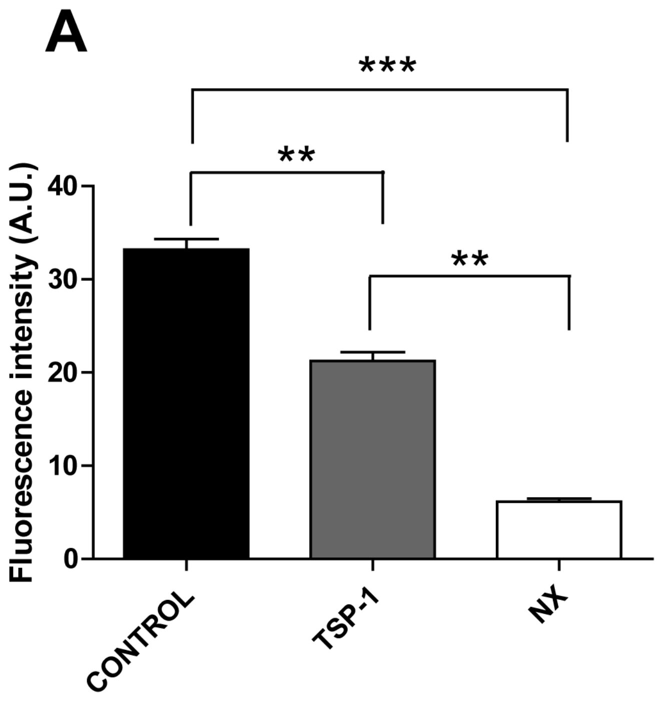

Supplementary Figure 3: Quantification of vascular density in tumor grown on the chick CAM. Fluorescent staining by SNA lectin in Figure 4 was quantified by counting in 4 random images from each treatment group. Results are presented as mean {plus minus} SEM from 3 independent experiments. The vessel density was significantly decreased in NX treated tumors compared to control ($p<0.001$) or TSP-1(0.0011). Comparisons between the treatments and controls were performed by using analysis of variances (ANOVA). ** $p<0.01$ ***$p<0.001$.

Primers and probes table

Gene Name	symbol	Accession number	species	Specific probe ID (ThermoFischer Scientific)
Angiopoietin 1	ANGPT1	NM_001146	H. Sapiens	Hs00375822_m1
Angiopoietin 2	ANGPT2	NM_001118887	H. Sapiens	Hs01048042_m1
CD36 molecule	CD36	NM_001030731	Gallus gallus	Gg03354931_m1
Fibroblast Growth Factor	FGF2	NM_002006	H. Sapiens	Hs00266645_m1
hypoxanthine-guanine phosphoribosyltransferase 1	HGPRT1	NM_000194	H. Sapiens	Hs02800695_m1
Integrin subunit beta 1	ITGB1	NM_001039254	Gallus gallus	Gg03357875_m1
Kinase Insert Domain Receptor	KDR (VEGFR2)	NM_001004368	Gallus gallus	Gg03346164_m1
Platelet Derived Growth Factor Beta polypeptide	PDGFB	NM_002608	H. Sapiens	Hs00966522_m1
Plasminogen Activator Urokinase	PLAU (uPA)	NM_002658	H. Sapiens	Hs01547054_m1
TIMP metallopeptidase inhibitor 1	TIMP1	NM_003254	H. Sapiens	Hs01092512_g1
TIMP metallopeptidase inhibitor 4	TIMP4	NM_003256	H. Sapiens	Hs00162784_m1
Vascular Endothelial Growth Factor A	VEGFA	NM_001025366	H. Sapiens	Hs00900055_m1

Gene Name	Symbol	Accession number	Species	Forward	Reverse	Probe
platelet-derived growth factor receptor, beta polypeptide	PDGFRB	XM_015293926	Gallus gallus	GGGACCTACGT CTGCAATGTCT	ACAAAGCCA CGCTCGATCA	TGAGGGCTAC CATGAGAA
tyrosine kinase with immunoglobulin-like and EGF-like domains 1	TIE1	XM_422400	Gallus gallus	AAGGTTGGTCCA TTGAGGAGAA	TCATGGTTACT CGGCAATTTCC	AACACCCTT CATGTCAAC
TEK tyrosine kinase, endothelial	TEK	XM_004949580	Gallus gallus	TGGAAAAGCCT CTCAACTGTGA	CAAACGACGGT CTTTCGTAAGG	TGAAGTGTATG ACCTAATGAG
